# Evaluating the Effects of Symptom Monitoring on Menopausal Health Outcomes: A Systematic Review and Meta-Analysis

**DOI:** 10.3389/fgwh.2021.757706

**Published:** 2021-12-03

**Authors:** Robin Andrews, Gabrielle Hale, Bev John, Deborah Lancastle

**Affiliations:** Faculty of Life Sciences and Education, School of Psychology, The University of South Wales, Wales, United Kingdom

**Keywords:** symptom monitoring, menopause, midlife, menopausal symptoms, MRC framework, health behaviour, e-health, women's health

## Abstract

Evidence suggests that monitoring and appraising symptoms can result in increased engagement in medical help-seeking, improved patient-doctor communication, and reductions in symptom prevalence and severity. To date, no systematic reviews have investigated whether symptom monitoring could be a useful intervention for menopausal women. This review explored whether symptom monitoring could improve menopausal symptoms and facilitate health-related behaviours. Results suggested that symptom monitoring was related to improvements in menopausal symptoms, patient-doctor communication and medical decision-making, heightened health awareness, and stronger engagement in setting treatment goals. Meta-analyses indicated large effects for the prolonged use of symptom diaries on hot flush frequencies. Between April 2019 and April 2021, PsychInfo, EMBASE, MEDLINE, CINAHL, Cochrane, ProQuest, PsychArticles, Scopus, and Web of Science were searched. Eighteen studies met the eligibility criteria and contributed data from 1,718 participants. Included studies quantitatively or qualitatively measured the impact of symptom monitoring on menopausal populations and symptoms. Research was narratively synthesised using thematic methods, 3 studies were examined via meta-analysis. Key themes suggest that symptom monitoring is related to improvements in menopausal symptoms, improved patient-doctor communication and medical decision-making, increased health awareness, and stronger engagement in goal-setting behaviours. Meta-analysis results indicated large effects for the prolonged use of symptom diaries on hot flush frequency: 0.73 [0.57, 0.90]. This review is limited due to the low number of studies eligible for inclusion, many of which lacked methodological quality. These results indicate that symptom monitoring has potential as an effective health intervention for women with menopausal symptoms. This intervention may be beneficial within healthcare settings, in order to improve patient-doctor relations and adherence to treatment regimes. However, findings are preliminary and quality assessments suggest high risk of bias. Thus, further research is needed to support these promising outcomes.

**Systematic Review Registration Number:** https://www.crd.york.ac.uk/prospero/display_record.php?, PROSPERO, identifier: CRD42019146270.

## Introduction

The menopause is diagnosed when over 12 months has passed since a woman last menstruated (1). Although cessation of menses is positively anticipated by some, the menopause, and the years prior, the perimenopause, can encompass a vast array of symptoms which can negatively impact quality of life (1, 2). These symptoms can be physiological in nature, including hot flushes and joint aches, whereas others impact psychological wellbeing, such as anxiety and low mood (1, 2).

Symptom monitoring has benefited numerous clinical populations, including patients with fibromyalgia, clinical depression, and cancer (3–5). Symptom monitoring involves recording symptoms using diaries, symptom questionnaires, or electronic health (e-health) instruments, including mobile apps or online symptom trackers; this can take place across time, or retrospectively at one or more given time points (6). Notable outcomes have included increased likelihood of accessing healthcare, heightened survival rates, and reductions in symptom prevalence and severity (3–5, 7, 8).

Despite broader literature demonstrating beneficial effects, symptom monitoring has not been formally evaluated as an intervention (3–5, 7, 8). According to the Medical Research Council's (MRC) framework for developing complex interventions, existing evidence for an intervention should ideally be collated into a systematic review to understand whether it is likely to be effective, whether it can be implemented into a research setting, and whether it can be widely implemented if the results are favourable (9). Should a review find evidence that an intervention is related to beneficial outcomes, the next evaluative phase should include an exploratory trial to measure the observed effects (9).

Thus, this review was designed according to three objectives: to assess whether symptom monitoring improves health outcomes relating to the menopause, e.g., changes in severity and reporting frequency of symptoms, or greater adherence to health-related behaviours; to evaluate mechanisms explaining why symptom monitoring influences health outcomes related to the menopause, e.g., whether it leads to greater health awareness or medical help-seeking; and, to categorise and compare the effects of different methods of symptom monitoring in order to inform best practise and intervention development.

The studies included in this review reported quantitative or qualitative data on the independent effects of symptom monitoring on menopausal symptoms, either as the intervention of interest or as a comparison or control intervention. Key outcomes included variances in symptom incidence or severity, and changes in health-related behaviour.

## Methodology

The methods used in this review are briefly summarised here, however it is recommended that the PROSPERO protocol be referred to for further details: registration number: CRD42019146270, available from: https://www.crd.york.ac.uk/prospero/display_record.php?ID=CRD42019146270. This review was developed in accordance with the PRISMA guidelines, and the Cochrane handbook for systematic reviews of interventions was consulted and the guidance followed (10).

### Search Strategy

Between April 2019 and August 2019, the following databases were searched PsycINFO, EMBASE, MEDLINE, CINAHL, Cochrane Library, ProQuest, PsycARTICLES, Scopus, and Web of Science. Updated searches took place in August 2020 and April 2021. Additional records were identified through forward and backward citation searches. Filters were used to focus the searches on English-language, female, adult-human participants. Date and geographical restrictions were not applied. Included studies had quantitatively or qualitatively assessed the independent effects of symptom monitoring on menopausal health outcomes.

Titles and abstracts were screened according to three preliminary criteria:

1. Participants included human adults, either mixed-gender or female-only. Mixed-gender research had to provide isolated data by gender.
2. The study collected data on the independent influence of symptom monitoring on health outcomes, either as the intervention of interest or as a comparison or control intervention, excluding research which instructed participants to monitor their symptoms in conjunction with a separate intervention.
3. The study explored common menopausal symptoms, excluding conditions which could impair an individual's ability to monitor their symptoms accurately, or could lead to fatality, hospitalisation, or were developed as a result of trauma.

**Figure 1:**
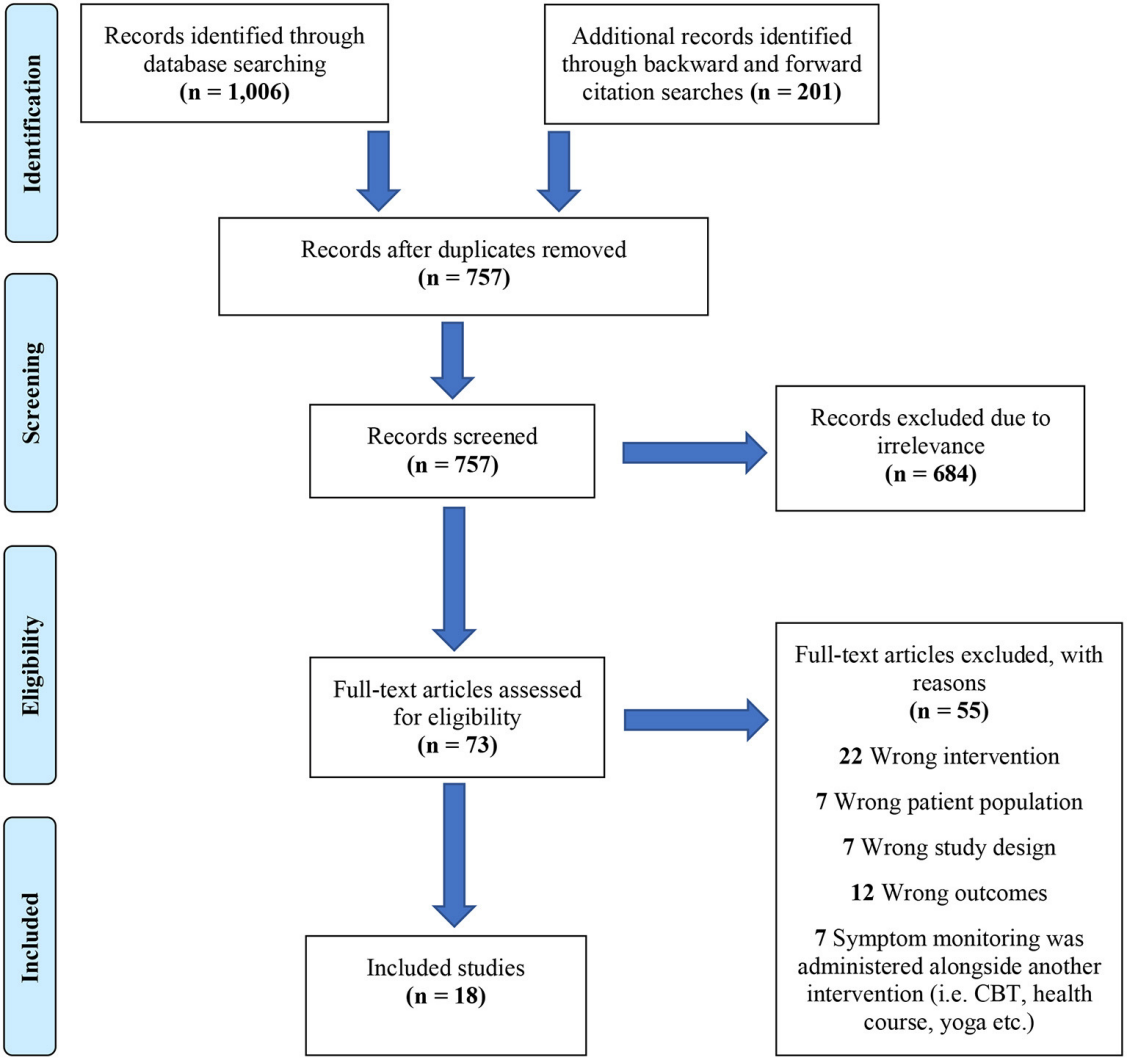
PRISMA flowchart of study selection process.

Full-text reports which appeared to meet the outlined criteria were screened independently by two authors. Prior to screening, authors engaged in a calibration process in order to enhance inter-reviewer consistency by screening 25 randomly selected studies. A Kappa statistic of: *κ*=0.757, *ρ*= 0.001 quantified agreement, indicating substantial inter-reviewer consistency (11). Any disagreements were discussed by authors. A PRISMA flowchart was produced to display the process of study selection, as well as reasons for exclusion (see Figure 1).

### Quality Assessment

For quantitative research, the National Institute for Health and Care Excellence (12) developed a checklist based around the Graphical Appraisal Tool for Epidemiological studies [GATE; (13)]. NICE (12) appropriated this checklist for evaluating health intervention studies. This checklist was deemed suitable for this review as it can be used to appraise the internal and external validity of various research designs.

An appraisal tool for qualitative research was used to assess studies with these characteristics (14). This checklist is based around characteristics of qualitative research which may affect a study's internal validity, i.e. whether the links between the data, interpretations, and conclusions are clearly defined (14).

Both checklists were amended according to each study under review; in cases where randomisation did not occur (i.e. in observational studies) judgements were made based on whether confounding variables may have influenced findings. This method has been implemented into the Cochrane risk of bias tool, hence why it was deemed appropriate (10).

### Data Analysis

A thematic synthesis provided a means of combining qualitative and quantitative information in a succinct manner. This meant that findings from all included studies could be simultaneously evaluated and compared. Key themes were identified by summarising the extracted results of each study, with contributions being made by 2 authors.

RevMan 5.3 software was used to compute a meta-analysis of comparable studies which used continuous data. Three studies described comparable study designs and symptom monitoring methods, pre-treatment and post-treatment data were combined, and the standardised mean difference and 95% confidence intervals (CIs) were computed. A random-effects model was used to compensate for variations within the selected studies. The classification of effect sizes was determined from Cohen's (15) conventional values of effect size which indicate: 0.20 = small effect size, 0.40 = medium effect size, and 0.60 = large effect size. Significance was set at *ρ* < 0.05.

Heterogeneity was tested using the I^2^ value. The interpretation of the observed I^2^ value was determined by The Cochrane Handbook of Systematic Reviews guidance which indicated that I^2^ <30% was considered mild heterogeneity, I^2^ >30% as moderate heterogeneity, I^2^ >50% as substantial heterogeneity and I^2^ >75% as considerable heterogeneity (16).

Due to differences in menopausal symptoms and symptom monitoring methods, populations and interventions varied, therefore results were predominantly synthesised narratively. A meta-analysis was conducted on three studies which had provided data on the impact of symptom diaries on menopausal hot flushes.

## Results

### Study Characteristics

Searches returned 18 studies which met the eligibility criteria and contributed data from 1,718 participants, see Figure 1 to view summary of study selection process.

Two studies involved the Women's Health Assessment Tool (WHAT), a symptom questionnaire relevant to midlife women (17, 18). Two studies included the Menopause Rating Scale (MRS), a questionnaire developed to evaluate menopausal symptom frequency, severity, and change (19, 20). Two studies explored The Computerised Symptom Capture Tool for Menopause [C-SCAT-M; (21, 22)]. The C-SCAT-M is a mobile app which features 54 menopausal symptoms, which were originally identified via the Seattle Women's Midlife Health Study (SWMHS). One study qualitatively explored the effects of daily self-weighing on perimenopausal weight changes (23). One study explored the MenoScores Questionnaire, the primary outcome was the hot flush scale (24). Four studies provided data on the effects of symptom diary use on perimenopausal symptoms, menopause-related headaches, sexual dysfunction, and incontinence (25–28). Six randomised controlled trails (RCTs) provided data on symptom-diary use and hot flush frequency (29–34). Three RCTs were evaluated via meta-analysis (30–32). See Table 1 to view a full summary of the characteristics of the included studies.

**Table 1:** Visual summary of study characteristics.

**Authors/** **geographical** ** region**	**Study design (sample size)**	**Menopause symptom(s)**	**Menopausal status (mean age)**	**Symptom monitoring method**	**Details of administration of symptom monitoring**	**Data relating to the effects of symptom monitoring**
Blümel et al. (19) Chile	Single-group pre-post (427)	Hot flushes, heart discomfort, sleep problems, muscle and joint pain, sexual problems, bladder problems, vaginal dryness, depressed mood, irritability, anxiety, physical and mental exhaustion	Pre/peri/postmenopausal (50.5)	Menopause Rating Scale (MRS)	Participants were surveyed once to determine a cut-off score on the MRS to indicate a need for treatment with HRT. Each symptom-specific item on the MRS was accompanied with another item which asked participants to indicate whether they thought that particular symptom required treatment.	After completing the MRS women were able to recognise when they needed medical help for their symptoms, supported by the items asking women if particular symptoms required treatment.
Berin et al. (29) Sweden	RCT- 2 conditions: resistance training vs usual activity (58)	Hot flushes	Postmenopausal (55.3)	Daily diaries recorded hot flush frequency.	Daily hot flush diaries were adhered to by both groups for 2 weeks at baseline, and then daily for up to 15 weeks.	From baseline to 15 weeks there was a non-significant 2% reduction in hot flushes in the usual activity control group which adhered to the symptom diary only. However, there was a significant 43.6% decrease in hot flushes in the resistance training group.
Borud et al. (32) Norway	RCT- 2 conditions: acupuncture with self-care vs self-care alone (399)	Hot flushes	Postmenopausal (53.8)	Diary recorded frequency and severity of hot flushes and sleep duration.	Daily diaries were administered to both participant groups for 2 weeks at baseline, and for 1 week at weeks 4, 8 and 12.	In both groups there were similar reductions in daily hot flush frequency and intensity, and small increases in hours of sleep per night.
Borud et al. (33) Norway	6- and 12- month follow up to an RCT- 2 conditions: acupuncture with self-care vs self-care alone (267)	Hot flushes	Postmenopausal (53.8)	Diary recorded daily frequency and severity of hot flushes and sleep duration.	Daily diaries were administered to both participant groups at weeks 4, 8, and 12. During the 6- and 12- month follow-ups, identical recordings were performed.	From baseline to six months, there were similar reductions in daily hot flush frequency and intensity among both groups.
Carpenter et al. (31) USA	RCT- 3 conditions: paced respiration vs breathing control vs care as usual (218)	Hot flushes	Peri/postmenopausal (52.96)	Electronic hot flush diaries for ≥24 h to ≤ 7 days (time determined by participant choice).	Hot flush frequency, severity, and bother (vasomotor symptoms), were prospectively recorded in diaries in real time by both participant groups at 2 weeks baseline and at 8 and 16 weeks	Paced respiration was not significantly more efficacious than breathing control or usual care for producing a 50 % reduction in hot flushes. Percentages achieving 50 % reduction in hot flashes from baseline to 16 weeks were: 38 % intervention, 29 % breathing control, and 22 % usual care control.
Hale et al. (27) Australia	Observational (24)	Breast tenderness, vasomotor symptoms, period changes	Perimenopausal (47)	Daily perimenopause diary	Participants used the perimenopause diary to record hot flush frequency and severity, breast tenderness, and menstruation across 4 menstrual cycles each (data on 98 cycles was collected in total).	Diaries show premenstrual increases in breast tenderness and VMS. Because hot flushes were related to menstrual cycles, the study hypothesised that these women may not benefit as much from hormone therapy, therefore diaries could help women make informed decisions on whether to use HRT.
Huang et al. (26) USA	Pilot RCT- 2 conditions: yoga therapy vs usual care (19)	Stress, urgency, or mixed-type urinary incontinence	Peri/postmenopausal (61.4)	7-day voiding diaries	Participants recorded each time they leaked urine and classified their leakage episodes as stress type (associated with coughing, sneezing, lifting, or physical activity), urgency-type (associated with a strong need or urge to void), or other-type (not associated with physical activity or with an urge to void).	The mean frequency of incontinence decreased by an average of 66% from baseline in the yoga therapy group and 13% in the control group. Stress incontinence frequency decreased by an average of 85% in the yoga therapy group, compared to a mean increase of 25% for the control group.
Irvin et al. (34) USA	RCT-3 conditions: relaxation response vs reading control vs symptom charting (33)	Hot flushes	Peri/postmenopausal (47.43)	Daily symptom diary measuring the frequency and intensity of hot flushes	Each subject completed a daily hot flush symptom diary form. Participants were instructed to record the frequency and intensity of daily hot flash symptoms using a Likert-type scale with values from 1 to 7, with 1 being mild and 7 being extremely severe.	All the groups had a decrease in flash frequency, but these did not reach statistical significance. The Relaxation group demonstrated a significant decrease in hot flush intensity. The Reading group demonstrated a small non-significant decrease while the control group demonstrated a small non-significant increase.
Ismail et al. (21) USA	Feasibility study (30)	54 menopausal symptoms, as indexed by the SWMHS, including hot flushes, sleeping problems, night sweats, and fatigue.	Peri/postmenopausal (40–60)	Computerised symptom capture tool for menopause (C-SCAT-M), a symptom heuristics application (app) for the iPad.	While completing the C-SCAT-M app women were asked to “think aloud”, and these thoughts were recorded and transcribed. Women completed the app using an iPad and they identified symptom clusters by drawing links between their current symptoms.	Most women stated that the final diagrams were very/extremely accurate depictions of their symptom clusters (77%). Participants were asked about their feelings in response to being requested to think about their symptoms while completing the app. Women said they felt “fine,” “surprised,” “sad,” “annoyed,” “depressed,” and “more aware about their symptoms.” Other women even reported finding solutions to their symptoms as they completed the app.
Lund et al. (24) Denmark	RCT- conditions: resistance training vs usual activity (66)	Hot flushes, day-and-night sweats, general sweating, sleep problems, emotional symptoms, memory changes, skin and hair symptoms, physical symptoms, abdominal symptoms, urinary and vaginal symptoms, sexual symptoms and tiredness.	Pre/peri/postmenopausal (54.7)	MenoScores questionnaire	All participants completed the MSQ after receiving it by email in study weeks 0, 3, 6, 8, 11 and 26.	The control group showed a trend of improvement, in particular in the hot flush scale, which may be explained by a regression to the mean. The acupuncture treatment reduced the hot flush, day-and-night sweats, general sweating, sleep problems, emotional symptoms, physical symptoms, and skin and hair scales after 5 weekly treatments. The intervention did not significantly reduce the remaining MSQ scales,
Muin et al. (25) Austria	Pilot single-arm non-randomised trail (30)	Low mood and sexual dysfunction	Peri/postmenopausal (53)	Sexual activity record (completed by participants only) and a sexual diary (completed by participants and their partners)	The sexual activity record and the sexual diary were administered for 4 weeks. The sexual activity record was used to document the time and date of sexual events. Participants and their partners recorded their current sexual satisfaction and/or fantasies, as well as anything else that might have interfered with their most recent sexual activity (i.e. partner's absence or illness) using the sexual diary.	A subjective improvement in communication of sexual problems was reported by 60% of participants; no participants reported any worsening of communication. This finding was not paralleled by objective measures taken using the Female Sexual Function Index (FSFI) and the Female Sexual Distress Scale (FSDS), neither of which had found statistically significant improvements. Significant improvements were found using the Hamilton Depression Scale (HDS), suggesting that participants had lower levels of depression after using the sexual diary.
Silvestrin et al. (17) USA	Prospective pilot study (110)	Hot flushes, depressed mood, anxiety, sexual dysfunction, vaginal dryness, bladder problems, breast pain	Peri/ postmenopausal (54.3)	Women's Health Assessment Tool (WHAT) (35-item patient self-reported questionnaire that assesses health conditions relevant to mid-life women).	The WHAT was administered through MyChart, an online patient portal system. One week prior to their annual well-woman visit participants were asked to complete the online WHAT questionnaire. After this visit patients were surveyed about their perceptions of using the WHAT questionnaire.	Most patients felt more prepared for their annual visit (69.7%), improvements in patient-doctor communication (69.8%) and that quality of care improved (68.4%) while clinicians reported streamlined patient visits and improved communication with patients. Most (71.1%) women “agreed” or “strongly agreed” that they would use the questionnaire again.
Silvestrin et al. (18) USA	Prospective pilot study (110)	Hot flushes, depressed mood, anxiety, sexual dysfunction, vaginal dryness, bladder problems, breast pain	Peri/ postmenopausal (54.3)	Women's Health Assessment Tool (WHAT) (35-item patient self-reported questionnaire that assesses health conditions relevant to mid-life women).	The WHAT was administered through MyChart, an online patient portal system. One week prior to their annual well-woman visit participants were asked to complete the online WHAT questionnaire.	There were 31 new diagnoses made during the well-woman visit, representing a 72.2% change in the diagnoses rate compared with the visits from 12 months prior (previous 12-month diagnoses: n = 18). Hot flushes had the greatest number of new diagnoses during the well-woman visit, closely followed by vulvovaginal atrophy and depression.
Stensland and Malterud (28) Norway	Single case study (1)	Headache	Menopausal (48)	Illness diary: Strength of headache, localisation, accompanying symptoms, situations where it occurred and medication.	The participant made notes in the diary for 1 month. Introduced to diary during first meeting and reviewed 4 weeks later (based on written material and conversation) and 4 months later when a clinical supervisor participated	After 1 month the patient had taken less medication and had fewer headaches. After 4 months she had not consulted a GP since her last appointment and had even fewer headaches than before. When asked about writing things down she said she had become more conscious, more reflective, and had more of a hold on self. She also felt calmer and had fewer sleep disturbances and used less medication.
Sternfeld et al. (30) USA	RCT- 2 conditions: exercise vs usual activity (248)	Hot flushes, depressed mood, anxiety, sleeping problems	Peri/ postmenopausal (55)	Daily diaries recorded hot flush frequency and bother.	Hot flush frequency and bother were recorded for 2 weeks at baseline and for 1 week at weeks 6 and 12. How flush bother was rated each day on a scale of 1-4	12 weeks of moderate intensity aerobic exercise did not reduce frequency or bother of VMS more than usual activity in initially sedentary women. At the end of week 12, changes in VMS frequency in the exercise group (mean change of −2.4/day, 95% CI −3.0, −1.7) and VMS bother (mean change of −0.5 on a 4 point scale, 95% CI −0.6, −0.4) were not significantly different from those in the control group (−2.6 VMS/ day, 95% CI −3.2, −2.0, p=0.43; −0.5 points, 95% CI −0.6, −0.4, p=0.75).
Su et al. (23) Taiwan	Qualitative (18)	Weight gain	Perimenopausal (51.5)	Daily self-weighing	Not administered during study, some participants discussed monitoring their weight daily during interviews	Several women measured their weight daily. If they had gained weight they reviewed all the possible reasons. Key themes derived from interviews included ‘enjoying my new life,’ ‘mastering self-monitoring of my health’ and ‘learning to communicate with the body’. Women enjoyed and mastered self-monitoring of their health, learned to communicate with their body and integrated new changes into their life: “I measure my weight every day to serve as a warning to myself. I often ask myself: did I eat too much recently? Otherwise, why did my weight increase? I think the body becomes healthier, I am enjoying life now.”
Woods et al. (22) USA	Single-group pre-post experimental design (30)	54 menopausal symptoms, as indexed by the SWMHS, including hot flushes, sleeping problems, night sweats, and fatigue.	Peri/ postmenopausal (40–60)	Computerised Symptom Capture Tool for Menopause (C-SCAT-M), a symptom heuristics application (app) for the iPad.	While completing the C-SCAT-M app women were asked to “think aloud”, and these thoughts were recorded and transcribed. Women completed the app using an iPad and they identified symptom clusters by drawing links between their current symptoms.	Most women (77%) stated that the final diagrams were very/extremely accurate in depicting their symptoms and their connexions. Women reported between 1 and 22 symptoms (median 11). Hot flushes, waking up during the night, night sweats, and early morning awakening were the most commonly reported symptoms. Women rated hot flushes as their most bothersome symptom, followed by waking up during the night and fatigue. They believed that hot flushes caused several symptoms, especially sleep disruption, and most could describe the time order of their symptoms. Women reported clusters consisting of 2 to 18 symptoms. Women also named each cluster based on their response to their symptoms (“really annoying”), the time of occurrence (“night problem”), and symptoms in the cluster (“hot flash”). They attributed their
						clusters to menopause and life demands. Many women requested a copy of their final symptom cluster diagram to discuss it with their health care providers.
Zangger et al. (20) Switzerland	Qualitative (3)	Hot flushes, sleeping problems, heart discomfort, depressed mood, irritability, anxiety, fatigue	Peri/ postmenopausal (46)	Menopause Rating Scale (MRS-II)	The patients filled in the MRS-II questionnaire prior to consultation with their physician. During the consultations, physicians transferred the patient's MRS responses into the digital ICF profile and discussed the goals to be achieved during therapy. The ICF profile organised the patient's MRS responses to depict their functional status. The tool also identified areas of poor functional capabilities related to their menopausal symptoms, and produced long, middle, and short-term goals for functional improvement.	From patients' perspective, filling in the ICF Categorical Profile helped to structure their goals but did not improve the overview of symptoms. Participants felt that filling in the ICF did not help identify their problems as they were already clear before. However, it helped them understand that their symptoms were connected. While filling in the ICF Profile, participants found thinking about their goals helped them handle their symptoms better. The participants stated that being able to visualise their symptoms had helped narrow them down and to see their limitations in a different light. All patients agreed that it was helpful to think about the goals they wanted to achieve and define them precisely as well as to define short-, middle- and long-term goals. Also, for all three patients the ICF Categorical Profile did not much change the decision process when discussing the treatment options. Though one patient stated that the visualisation helped to get an overview over the priorities of the treatment, she also said that the physician's opinion was most important for her to decide which treatment was best. The other two patients said that the priorities for treating their symptoms had been clear before, yet the ICF Categorical Profile had helped them to decide what treatment they wanted.

### Quality Assessment

Few studies were judged to have adequate validity, with all but 4 being considered unsatisfactory (30–33). See Table 2 to view quality assessment summary ranked from lowest to highest quality.

**Table 2:** Quality assessment summary.

**References**	**Study design**	**NICE quality checklist**	**Internal validity**	**External validity**	**Overall assessment**	**Notes**
Ismail et al. (21)	Qualitative	Qualitative checklist	-	-	-	• No justification for not triangulating data. • Small sample size. • Study design may have been vulnerable to demand characteristics/ desirability bias. • Did not describe role of researchers, nor methods of analysing the data.
Silvestrin et al. (17)	Observational/ cross sectional	Quantitative checklist	-	-	-	• Study design may have been vulnerable to demand characteristics/ desirability bias • Recruited from 3 clinics. • Sample was not ethnically diverse. • Did not use a control group to assess effect sizes.
Silvestrin et al. (18)	Observational/ cross sectional	Quantitative checklist	-	-	-	• Used before and after data from the same participants instead of a control group • Recruited from 3 clinics • Sample was not ethnically diverse.
Stensland and Malterud (28)	Case-study	Qualitative checklist	-	-	-	• Did not present data systematically. • Did not involve methods of triangulation • Did not discuss in detail reduction in medication use/ symptoms.
Woods et al. (22)	Qualitative	Quantitative checklist	-	-	-	• Study design may have been vulnerable to demand characteristics/ desirability bias • Objectives and a design were not clearly defined • Lack of triangulation was not justified but would have been useful to minimise bias.
Zangger et al. (20)	Qualitative	Qualitative checklist	-	-	-	• Number of researchers involved in transcript analysis was unclear • The interview setting/ context was unclear. • Results were not systematically reported.
Irvin et al. (34)	RCT	Quantitative checklist	-	-	-	• A small participant sample was used, therefore the study was not sufficiently powered. • Lack of blinding and allocation concealment. • Intervention exposure time may have been too long, many participants dropped out as they found 7 weeks of symptom charting too intrusive. • Did not include standard deviations in outcome data.
Blumel et al. (19)	Observational/ cross sectional	Quantitative checklist	+	-	-	• Participants were recruited from a single clinic. • Used highly subjective measures rather than diagnostic tests or filled prescriptions.
Muin et al. (25)	Non-randomised trial	Quantitative checklist	+	-	-	• Participants were treatment seeking so may not appropriately represent women with sexual dysfunction. • Used a small participant sample- therefore may have been underpowered to detect significant effects. • Did not include randomisation which would have shown a more rigorous effect for the sexual diary and sexual activity record.
Lund et al. (24)	RCT	Quantitative checklist	+	-	-	• The intervention group had regular meetings with GPs. This may have increased the likelihood of experimenter bias or demand characteristics/ social desirability bias. • Participants were recruited using adverts in acupuncture authorities, which meant these participants were self-selecting and likely to be inclined toward the intervention (acupuncture).
						• It is not clear whether reductions in hot flushes in the control group reached statistical significance.
Su et al. (23)	Qualitative	Qualitative checklist	+	-	+	• No methods of triangulation described.
Berin et al. (29)	RCT	Quantitative checklist	+	-	+	• The intervention group had regular meetings with physiotherapists. This may have increased the likelihood of experimenter bias. • Exposure time may have been too long, as many control participants did not consistently complete symptom diaries over 15 weeks. • Most incomplete diaries belonged to the control group (10/14), which could have increased the risk of bias in the results.
Huang et al. (26)	RCT	Quantitative checklist	+	-	+	• Participants were not blinded to allocation, which could have affected results. • A small participant sample was used, therefore the study was not well powered. • There were varying characteristics between groups, but it is unclear whether these were adjusted for.
Hale et al. (27)	Observational	Quantitative checklist	++	-	+	• Long list of symptoms to monitor each day, may have increased likelihood of retrospective recording and data inaccuracies. • Small sample size.
Carpenter et al. (31)	RCT	Quantitative checklist	+	+	+	• Participants were offered a cash incentive, which could have increased response bias. • Non-blinded staff carried out quality-check visits to see if the intervention was being adhered to correctly, this could have led to experimenter bias, demand characterises, or desirability bias. • Wait-list control could have been more appropriate to conceal allocation.
Sternfeld et al. (30)	RCT	Quantitative checklist	++	+	+	• Does not discuss whether the symptom diary or participation may have elicited the reductions in symptoms across all groups. • Exercise group spent time with a trained exercise practitioner which could have led to social desirability bias.
Borud et al. (32)	RCT	Quantitative checklist	++	+	+	• It was not possible to blind participants to exposure. • Participants applied to the study via adverts, which could indicate self-selection bias. • Participants had a positive attitude toward the key intervention (acupuncture), therefore contamination may have been more likely.
Borud et al. (33)	RCT	Quantitative checklist	++	+	+	• A 6 and 12 month follow up of the above. • High risk of exposure to factors which could have affected the results (i.e. HRT use, over the counter medication, exercise or behavioural interventions).

*Key*.

*++ = All or most of the checklist criteria have been fulfilled, where they have not been fulfilled the conclusions are very unlikely to alter*.

*+ = Some of the checklist criteria have been fulfilled, where they have not been fulfilled, or not adequately described, the conclusions are unlikely to alter*.

*- = Few or no checklist criteria have been fulfilled and the conclusions are likely or very likely to alter*.

### Qualitative Synthesis

#### Themes

Five themes were identified, one was related to physiological changes following adherence to symptom monitoring and encompassed variations in symptom frequency and severity regarding hot flushes, urinary incontinence, migraines, sexual dysfunction, and depression; the other four themes encompassed health-related behavioural changes and included improvements in patient-doctor communication, shared decision-making, health and symptom awareness, and goal setting.

#### Physiological Changes

##### Hot Flushes

Reductions in hot flush frequency were present among studies which involved the use of symptom diaries (24, 30–33). Four randomised trials included before and after data on the effects of symptom diaries on vasomotor symptoms (VMS) and reported significant reductions in hot flush frequency scores (30–33). Diary studies with smaller sample sizes also reported reductions in VMS, although these findings were non-significant (29, 34).

##### Urinary Incontinence

Huang et al. (26) used voiding diaries to research symptom change and frequency among 19 menopausal women with urinary incontinence partaking in a yoga therapy intervention. All participants kept 7-day voiding diaries where they recorded each time they leaked urine. After 6 weeks, total incontinence frequency had reduced by 13% in the control group which used the voiding diary alone.

##### Migraines

Stensland and Malterud (28) described a perimenopausal patient presenting with worsening migraine and muscle pain. This patient was advised to compile an illness diary for 4 weeks, detailing the strength of her headache, its localisation, accompanying symptoms, situations where it had occurred, and medications used (28). After 4 weeks of diary use the patient reported using half as much medication as she had the month prior (28). At four-month follow-up the patient stated that her symptoms had improved even more so, and attributed these improvements to her becoming more aware and reflective of her health through the symptom-diary (28).

##### Sexual Functioning and Depression

Muin et al. (25) studied 30 menopausal participants diagnosed with sexual dysfunction, who were asked to keep a sexual diary for 4 weeks. Sixty percent of participants reported subjective improvements in sex life and sexual communication and significant improvements were found using the Hamilton Depression Scale (HDS).

#### Behavioural Changes

##### Patient-Doctor Communication

Two studies explored the WHAT menopause symptom questionnaire among menopausal patients attending a well woman clinic (17, 18). These studies reported that the WHAT had improved patient-doctor communication from both the clinicians' and patients' perspectives, which led to a rise in the number of diagnoses made, especially for sensitive conditions including vaginal dryness, sexual dysfunction and depression (17, 18).

Ismail and colleagues (21) evaluated the C-SCAT-M app, which enables users to appraise their menopause symptoms and generate a report. Participants were asked to voice their thoughts and experiences whilst completing the app, and many requested a copy of their symptom report to share with their physician, commenting that it would be a useful communication aid (21).

##### Shared Decision-Making

Two studies evaluating the MRS found it improved patients' efficacy in making medical treatment decisions (19, 20).

Hale et al. (27) employed symptom-diaries to explore whether menopausal symptoms were related to the menstrual cycle. Diaries showed premenstrual increases in breast tenderness and VMS, therefore authors hypothesised that symptom diaries could help women identify when cyclical HRT or non-hormonal treatments might be more beneficial than continuous HRT (27).

##### Health & Symptom Awareness

During Zangger et al.'s (20) study, one participant stated that being able to see her symptoms visually represented on the MRS had enabled her to recognise that they were connected. In Ismail and colleagues (21) study, participants found solutions to their symptoms as they completed the C-SCAT-M app. The researchers proposed that heightened symptom awareness had prompted participants to reflect upon strategies to alleviate their symptoms.

Su et al. (23) evaluated the weight loss experiences of 18 obese perimenopausal women who had been diagnosed with metabolic syndrome. Findings demonstrated that awareness of weight gain was a factor that motivated perimenopausal women to change their lifestyle habits (23). Participants expressed that they had become more aware of their health via daily self-weighing, which made them feel better able to “communicate with their body's voice” (23).

##### Goal Setting

Another finding to emerge from Zangger et al.'s (20) study was related to goal setting. After completing the MRS, women were able to restructure their symptom scores into short-, middle, and long-term health goals. Another study conducted by Woods and colleagues (22) evaluated the C-SCAT-M. This research focused on the app's ability to allow women to communicate their heuristics related to menopausal symptom attributions (22). By using the C-SCAT-M, women reported their symptoms, grouped them into clusters, specified the order of occurrence, gave the clusters meaningful names, and identified relationships between symptoms. The authors speculated that by appraising, naming, prioritising, and grouping their symptoms, participants gleaned a greater understanding of what they were hoping to achieve in terms of treatment.

### Comparing Symptom Monitoring Methods

Monitoring symptoms across time appeared to be useful for reducing symptom frequencies, as well as medication usage, and this effect may be explained by heightened health and symptom awareness (24–26, 28–34). Patient-doctor communication was facilitated by tools which allowed users to define the totality of their symptoms from an exhaustive inventory (17, 18, 21). Medical decision-making was facilitated by utilising symptom appraisal tools in conjunction with items assessing medication need, or digital ICF profiles, thus suggesting that input from medical systems is vital for unlocking the tool's decision-making capabilities (19, 20). Medical decision-making may also be improved by menstrual diaries, as they can allow patients and practitioners to understand whether VMS are related to the menstrual cycle or menopause (27). An illness diary, symptom appraisal tools, and daily self-weighing were related to improvements in health and symptom awareness, and this increased awareness was related to symptom improvements and changes in health-related behaviours (20, 21, 23, 28). Finally, symptom appraisal tools which allowed the user to compare their symptoms and corresponding severities supported setting treatment goals (20, 22).

### Meta-Analysis

#### Included Studies

Three RCTs were deemed appropriate for evaluation via quantitative synthesis. These studies included continuous data measuring the independent effects of prolonged symptom diary use on VMS frequency (30–32). Using a random effects model, the standardised mean difference and statistical heterogeneity with Chi^2^ and I^2^ figures were calculated.

#### Excluded Studies

Three other studies measured the independent effects of prolonged symptom diary use on VMS (29, 33, 34). One was excluded because it used a small participant sample (*N* = 11) in the symptom diary arm and failed to provide standard deviations for mean frequency of VMS following symptom diary use (34).

Berin et al.'s (29) study was excluded from the main meta-analysis because a disproportionate amount of missing data was attributed to the control group. The control group provided before and after data from participants who had been using VMS diaries for 15 weeks, however 35% of these participants did not consistently complete diaries. This meant the analysis likely underestimated reductions in VMS (29). To understand the impact of excluding this study on the overall effect of symptom monitoring, a meta-analysis was conducted with Berin et al.'s (29) data included and it suggested a large effect size (0.61, 95% CI 0.34 to 0.88) with the difference being significant in favour of post symptom-diary use (Z = 4.45, *p* = 0.001). However, heterogeneity was substantial (I^2^ = 60%).

A third study was excluded which depicted a 6- and 12-month follow-up of a study which is included in the present meta-analysis (33). The later study increased the heterogeneity statistic (I^2^ = 78%), therefore the earlier study by Borud et al. (32) was chosen for inclusion.

#### Reduction in Symptom Frequency

The three included studies involved a total of 303 participants at baseline, and 296 participants post-intervention. Each study reported continuous data for VMS frequencies. Two studies included mean differences in VMS frequency following symptom diary use therefore mean VMS frequency following symptom-diary use was calculated by subtracting the post-intervention mean difference from the pre-intervention mean (30, 32). Borud et al. (32) reported mean frequency of VMS at baseline and the standard deviation (13.1, 4.9) and mean change in VMS frequency following symptom diary use and the standard deviation (9.4, 3.7). Sternfeld et al. (30) reported confidence intervals instead of standard deviations for baseline VMS frequency (8.0 [7.3, 8.7]) and mean change in VMS frequency following symptom diary use (−2.6 [−3.2, −2.0]), therefore confidence intervals for baseline and post-intervention measures were recalculated into standard deviations (4.26, 3.56). Carpenter et al. (31) reported mean frequency of VMS and standard deviations pre (7.31, 4.04) and post prolonged symptom-diary use (4.76, 3.5).

**Figure 2:**
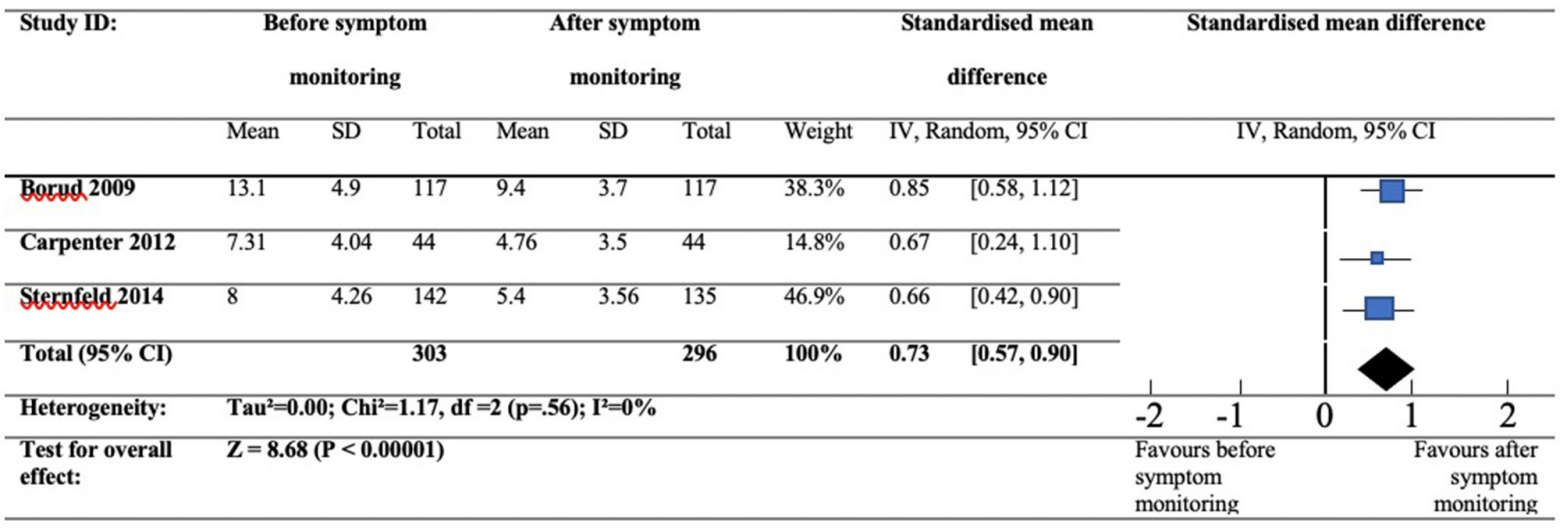
*F*orest plot showing hot flush frequency at baseline and post-symptom diary use.

The standardised mean difference for symptom frequency suggested a large effect size (0.73, 95% CI 0.57 to 0.90) and the difference was significant in favour of post symptom-diary use (Z = 8.68, *p* = 0.001). Heterogeneity was absent (I^2^ = 0%). See Figure 2 to view a forest plot detailing the effects of symptom diaries on hot flush frequency scores.

## Discussion

This review investigated whether symptom monitoring could yield improvements in menopausal symptoms and health-related behaviours. This suggests that symptom monitoring has potential as an effective intervention. Systematic searches returned 18 studies which contributed data from 1,718 participants. Methods which allowed patients to track their symptoms across time were related to symptom improvements and heightened health awareness, whereas tools which enabled patients to appraise their symptoms at any given time point enhanced health awareness, treatment goal setting, patient-doctor communication, and shared decision-making. A meta-analysis suggested large effects for the prolonged use of symptom diaries on VMS frequencies: 0.73 [0.57, 0.90]. In regard to the MRC framework, these results suggest that the effects of prolonged symptom monitoring be further measured via an exploratory trial; and should symptom monitoring be implemented within the wider community, it is recommended that symptom monitoring and appraisal methods should include an exhaustive array of symptoms and be used in conjunction with organised health systems in order to reap potential benefits in patient doctor communication, medical decision-making, and treatment goal setting (9).

The key finding from this review was that tracking symptoms could facilitate improvements (24–26, 28, 30–34). This is supported by the meta-analysis which assessed VMS frequency scores at baseline and after prolonged symptom diary use; results favoured post-diary use and showed large effects for reducing VMS (30–32). This result has been demonstrated by prior studies which have shown symptom reductions within alternative patient populations, suggesting that the beneficial effects of symptom monitoring are transferable across multiple ailments (5, 7, 8).

Symptom monitoring was also associated with improvements in health-related behaviours, including heightened awareness (21–23, 28). Ismail et al. (21) reported that women found solutions to their menopausal symptoms as they completed the C-SCAT-M, suggesting that symptom appraisal had brought the participant's health to the forefront of their minds, which prompted them to reflect, consider remedies, and invoke behavioural changes (21). A similar result was reported in a case-study whereby a patient reported reductions in medication use and headache symptoms following use of a symptom diary; the patient attributed these improvements to the symptom diary heightening her awareness, which prompted her to amend her health behaviours accordingly (28).

The MRS and the C-SCAT-M were related to treatment goal setting (20, 22). Goal setting is a facet of Chewning and Sleath's (35) client-centred model of medical decision-making and management. This model depicts how the patient and physician could work together to carry out the following practises: identify treatment goals, choose treatment options, monitor symptoms and evaluate treatments, and revise treatments if problems occur.

Symptom monitoring methods could support this model, tools such as the MRS and the C-SCAT-M can help patients prioritise symptoms and identify the improvements they expect to see in the future, and this process can facilitate decision-making and patient-doctor communication. This theory corresponds with additional themes from this review, which found evidence that symptom monitoring could be useful for improving patient-doctor communication and facilitating decisions to use HRT and seek timely medical help (17–21, 27).

### Limitations and Future Directions

This review is limited by the low number of studies identified for inclusion, many of which were lacking methodological quality. Moreover, only three studies were integrated into the meta-analysis, meaning a funnel plot was not computed and risk of bias could not reliably be ascertained (36). Meta-analyses with low study inclusions are notorious for overestimating effect sizes (37).

To the author's knowledge, no other meta-analyses of this type have been conducted before, therefore this review provides a foundation for subsequent estimations of the effects of symptom diaries in symptom reduction. Numerous studies have given evidence that symptom monitoring could yield symptom improvements for a number of conditions (5, 7, 8). Therefore, future reviews should assess prolonged symptom monitoring on alternative conditions and symptoms.

An unexpected observation was that authors appeared to consider symptom monitoring as a benign “control” activity (29–32). Only two RCTs acknowledged the symptom reductions observed within the control groups, and neither considered whether symptom monitoring contributed to these reductions, despite symptom monitoring being the only activity control participants had been instructed to adhere to (24, 33). While Borud et al. (33) speculated that the improvements were due to “the therapeutic effects of research participation”, Lund et al. (24) posited that the reductions could be attributed to a regression toward the mean. This might be plausible given the non-significant reductions seen in studies with smaller sample sizes (29, 34). Although it is worth noting that all RCTs included in this review reported some degree of improvement within the control groups, and the majority reported significant effects. Furthermore, data from Berin et al.'s (29) research were excluded from the meta-analysis, as this study reported that 35% of participants did not consistently complete symptom diaries, suggesting reductions in VMS were likely to be underestimated (29). However, to understand whether this exclusion impacted the main review findings, a meta-analysis was conducted including Berin et al.'s (29) data and it continued to support the key result demonstrating large effects for the prolonged use of symptom diaries on VMS frequencies (0.61, 95% CI 0.34 to 0.88). These observations indicate that symptom monitoring can induce clinical benefits, which authors neither expect nor acknowledge, suggesting that there is an unrealised potential for monitoring to be implemented as a useful health intervention.

Further to this, in the included RCTs symptom monitoring was not intended as an intervention and therefore do not include non-symptom monitoring arms. Thus, it is not possible to discern whether any observed reduction in symptoms were an effect of symptom monitoring via diaries or due to alternative mechanisms (e.g., passage of time), although it would be anticipated that such mechanisms would also be in play in any intervention group. The MRC framework proposes that should reviewed literature suggest an intervention has potential benefits, the next phase to evaluate it should involve an exploratory trial (9). Therefore, future research should explore the effects of symptom monitoring as a standalone intervention for menopausal symptoms against a control intervention in which monitoring is not included. Such a design has previously been used in fertility research, which notably demonstrated that symptom monitoring was associated with improvements in anxiety and depression ratings (38).

However, evidence from the reviewed studies suggest that monitoring might be associated with increased attrition rates; Irvin et al. (34) reported that participants allocated into the monitoring group found symptom charting intrusive, whereas Berin et al. (29) mentions incomplete diaries in the control group. These factors may be indictive of negative consequences and could have an impact on the outcomes measured; therefore, further research should aim to understand the harms associated with symptom monitoring, as well as the benefits.

## Conclusions

This review suggests that symptom monitoring and appraisal methods are effective for reducing menopausal symptoms, and improving health awareness, shared decision-making, patient-doctor communication, and treatment goal setting. In line with the MRC framework, this suggests that symptom monitoring is likely to be an effective intervention (9). However, these findings are preliminary, and few studies were identified for inclusion, many of which lacked methodological quality, suggesting more research is needed to establish symptom monitoring as a useful intervention. A key finding was that prolonged symptom monitoring is effective for symptom reduction, a result which should further be investigated via an exploratory trial, in accordance with the methods established by the MRC framework (9). In terms of implementing symptom monitoring within the wider community, these results support that symptom appraisal tools should be used in conjunction with organised health systems to unlock potential improvements in decision-making, goal setting, and patient-doctor communication. Ultimately, the findings described here could provide foundational data to researchers wishing to unravel the effects of symptom monitoring, or develop a simple, cost-effective intervention which may have significant implications on menopausal health outcomes.

## Data Availability Statement

The raw data supporting the conclusions of this article will be made available by the authors, without undue reservation.

## Author Contributions

RA was responsible for conceptualising this review's design, collecting, analysing, and interpreting the data, and drafting the final article. GH contributed to the acquisition, analysis and interpretation of data, and critically revised and approved the article for publication. BJ and DL were responsible for conceptualising the study design, critically appraising the article for important intellectual content, and final approval for publication. All authors contributed to the article and approved the submitted version.

## Funding

This research is part of a PhD thesis which is funded by the University of South Wales, Knowledge Economy Skills Scholarships [KESS 2, a pan-Wales operation supported by European Social Funds (ESF) through the Welsh Government], and Health & Her. KESS-2 (grant number: c80815 KESS 2).

## Conflict of Interest

The authors declare that the research was conducted in the absence of any commercial or financial relationships that could be construed as a potential conflict of interest.

## Publisher's Note

All claims expressed in this article are solely those of the authors and do not necessarily represent those of their affiliated organizations, or those of the publisher, the editors and the reviewers. Any product that may be evaluated in this article, or claim that may be made by its manufacturer, is not guaranteed or endorsed by the publisher.

## References

[1] Dalal PK Agarwal M. Postmenopausal syndrome. Indian J Psychiatry. (2015) 6:S222. 10.4103/0019-5545.161483 26330639 PMC4539866

[2] Constantine GD Graham S Clerinx C Bernick BA Krassan M Mirkin S . Behaviours and attitudes influencing treatment decisions for menopausal symptoms in five European countries. Post Reproductive Health. (2016) 3:112–22. 10.1177/2053369116632439 26895640 PMC5019289

[3] Shafran R Gyani A Rostron J Allen S Myles-Hooton P Allcott-Watson H . Translating the intention to seek treatment into action: does symptom monitoring make a difference? results from a randomized controlled trial. Behav Cogn Psychother. (2019) 47:114–28. 10.1017/S1352465818000498 30136644

[4] Basch E Deal AM Kris MG Scher HI Hudis CA Sabbatini P . Symptom monitoring with patient-reported outcomes during routine cancer treatment: a randomized controlled trial. J Clin Oncol. (2016) 3:557–65. 10.1200/JCO.2015.63.0830 26644527 PMC4872028

[5] Collinge W Yarnold P Soltysik R. Fibromyalgia symptom reduction by online behavioral self-monitoring, longitudinal single subject analysis and automated delivery of individualized guidance. N Am J Med Sci. (2013) 9:546. 10.4103/1947-2714.118920 24251273 PMC3818828

[6] Ben-Zeev D McHugo GJ Xie H Dobbins K Young MA. Comparing retrospective reports to real-time/real-place mobile assessments in individuals with schizophrenia and a nonclinical comparison group. Schizophr Bull. (2012) 3:396–404. 10.1093/schbul/sbr171 22302902 PMC3329976

[7] Khakbazan Z Taghipour A Roudsari RL Mohammadi E. Help seeking behavior of women with self-discovered breast cancer symptoms: a meta-ethnographic synthesis of patient delay. PLoS ONE. (2014) 12:e110262. 10.1371/journal.pone.0110262 25470732 PMC4254513

[8] Devineni T Blanchard EB. A randomized controlled trial of an internet-based treatment for chronic headache. Behav Res Ther. (2005) 43:277–92. 10.1016/j.brat.2004.01.008 15680926

[9] O'Cathain A Croot L Duncan N N Rousseau Sworn K Turner KM . Guidance on how to develop complex interventions to improve health and healthcare. BMJ open. (2019) 9:e029954. 10.1136/bmjopen-2019-029954 31420394 PMC6701588

[10] Higgins JP Thomas J Chandler J Cumpston M Li T Page MJ . (2019) Cochrane handbook for systematic reviews of interventions, second ed. Chichester (UK): John Wiley and Sons. 10.1002/9781119536604 PMC10284251 31643080

[11] Landis JR Koch GG. The measurement of observer agreement for categorical data. Biometrics. (1977) 1:159–74. 10.2307/2529310 843571

[12] National Institute for Health and Care Excellence (NICE). Quality Appraisal Checklist: Quantitative Intervention Studies, Methods for the Development of NICE Public Health Guidance (3rd edition). (2019). Available online at: https://www.nice.org.uk/process/pmg4/chapter/appendix-f-quality-appraisal-checklist-quantitative-intervention-studies, 2012 (accessed 13 August 2019).

[13] Jackson R Ameratunga S Broad J Connor J Lethaby A Robb G . The GATE frame: critical appraisal with pictures. BMJ Evid Based Med. (2006) 2:35–8. 10.1136/ebm.11.2.35 17213070

[14] National Institute for Health and Care Excellence (NICE). Quality Appraisal Checklist: Qualitative Studies, Methods for the Development of NICE Public Health Guidance (3rd edition). (2019). Available online at: https://www.nice.org.uk/process/pmg4/chapter/appendix-h-quality-appraisal-checklist-qualitative-studies, 2012 (accessed 13 August 2019).

[15] Cohen J. (1962) The statistical power of abnormal-social psychological research: a review. J Abnorm Psychol. 65:145. 10.1037/h0045186 13880271

[16] Higgins JP Thompson SG. Quantifying heterogeneity in a meta-analysis. Stat Med. (2002) 11:1539–58. 10.1002/sim.1186 12111919

[17] Silvestrin TM Steenrod AW Coyne KS Gross DE Esinduy CB Kodsi AB . An approach to improve the care of mid-life women through the implementation of a Women's Health Assessment Tool/Clinical Decision Support toolkit. Women's Health. (2016) 5:456–64. 10.1177/1745505716664742 27558508 PMC5373265

[18] Silvestrin TM Steenrod AW Coyne KS Gross D Esinduy C Kodsi A . Outcomes of Implementing the Women's Health Assessment. Tool, and Clinical Decision Support Tool Kit Women's Health. (2016) 3:313–23. 10.2217/whe.16.3 27188377 PMC5384517

[19] Blumel JE Arteaga E Parra J Monsalve C Reyes V Vallejo MS . Decision-making for the treatment of climacteric symptoms using the Menopause Rating Scale. Maturitas. (2018) 111:15–9. 10.1016/j.maturitas.2018.02.010 29673828

[20] Zangger M Poethig D F Meissner von Wolff M Stute P. Linking the menopause rating scale to the International classification of functioning, disability and health–A first step towards the implementation of the EMAS menopause health care model. Maturitas. (2018) 118:15–9. 10.1016/j.maturitas.2018.10.003 30415750

[21] Ismail R Linder LA MacPherson CF Woods NF. Feasibility of an iPad application for studying menopause-related symptom clusters and women's heuristics. Inform Health Soc Care. 3:247–66. 10.3109/17538157.2015.1008489 26161593

[22] Woods NF Ismail R Linder LA Macpherson CF. Midlife women's symptom cluster heuristics: evaluation of an iPad application for data collection. Menopause. (2015) 10:1058. 10.1097/GME.0000000000000429 25803668 PMC4580486

[23] Su MC Lin HR Chu NF Huang CH Tsao LI. Weight loss experiences of obese perimenopausal women with metabolic syndrome. J Clini Nurs. (2015) 24:1849–59. 10.1111/jocn.12806 25753923

[24] Lund KS Siersma V Brodersen J Waldorff FB. Efficacy of a standardised acupuncture approach for women with bothersome menopausal symptoms: a pragmatic randomised study in primary care (the ACOM study). BMJ Open. (2019) 1:e023637. 10.1136/bmjopen-2018-023637 30782712 PMC6501989

[25] Muin DA Wolzt M Rezaei SS Tremmel-Scheinost M Salama M Fuchs C . Effect of sexual diary keeping and self-evaluation on female sexual function and depression: a pilot study, Eur J Contracept Reprod Health Care. (2016) 2:141–9. 10.3109/13625187.2015.1074676 26290038

[26] Huang AJ Jenny HE Chesney MA Schembri M Subak LL. A group-based yoga therapy intervention for urinary incontinence in women: a pilot randomized trial. Female Pelvic Med Reconstr Surg. (2014) 3:147–54. 10.1097/SPV.0000000000000072 24763156 PMC4310548

[27] Hale GE Hitchcock CL Williams LA Vigna YM Prior JC. Cyclicity of breast tenderness and night-time vasomotor symptoms in mid-life women: information collected using the Daily Perimenopause Diary. Climacteric. (2003) 2:128–39. 10.1080/cmt.6.2.128.139 12841883

[28] Stensland P Malterud K. Unravelling empowering internal voices- a case study on the interactive use of illness diaries. Fam Pract. (2001) 4:425–9. 10.1093/fampra/18.4.425 11477051

[29] Berin E Hammar M Lindblom H Lindh-Åstrand L Rubér M Holm AC. Resistance training for hot flushes in postmenopausal women: A randomised controlled trial. Maturitas. (2019) 126:55–60. 10.1016/j.maturitas.2019.05.005 31239119

[30] Sternfeld B Guthrie KA Ensrud KE LaCroix AZ Larson JC Dunn AL . Efficacy of exercise for menopausal symptoms: a randomized controlled trial. Menopause. (2014) 4 330. 10.1097/GME.0b013e31829e4089 23899828 PMC3858421

[31] Carpenter JS Burns DS Wu J Otte JL Schneider B Ryker K . Paced respiration for vasomotor and other menopausal symptoms: a randomized, controlled trial. Journal of general internal medicine. (2013) 2:193–200. 10.1007/s11606-012-2202-6 22936289 PMC3614127

[32] Borud EK Alraek T White A Fonnebo V Eggen AE Hammar M . The acupuncture on hot flushes among menopausal women (ACUFLASH) study, a randomized controlled trial. Menopause. (2009) 3:484–93. 10.1097/gme.0b013e31818c02ad 19423996

[33] Borud EK Alraek T White A Grimsgaard S. The Acupuncture on Hot Flashes Among Menopausal Women study: observational follow-up results at 6 and 12 months. Menopause. (2010) 2:262–8. 10.1097/gme.0b013e3181c07275 20009958

[34] Irvin JH Domar AD Clark C Zuttermeister PC Friedman R. The effects of relaxation response training on menopausal symptoms. J Psychosom Obstet Gynaecol 4. (1996) 202–7. 10.3109/01674829609025684 8997686

[35] Chewning B Sleath B. Medication decision-making and management: a client-centered model, Social science and medicine. (1996) 3:389–98. 10.1016/0277-9536(95)00156-5 8658233

[36] Lau J Ioannidis JP Terrin N Schmid CH Olkin I. The case of the misleading funnel plot. BMJ. (2006) 7568:597–600. 10.1136/bmj.333.7568.597 16974018 PMC1570006

[37] Seide SE Röver C Friede T. Likelihood-based random-effects meta-analysis with few studies: empirical and simulation studies. BMC Med Res Methodol. (2019) 1:16. 10.1186/s12874-018-0618-3 30634920 PMC6330405

[38] Ockhuijsen HA van den Hoogen M Eijkemans Macklon N Boivin J. Clarifying the benefits of the positive reappraisal coping intervention for women waiting for the outcome of IVF. Human Reproduct. (2014) 29:2712–2718. 10.1093/humrep/deu253 25316451

